# Inequalities in COVID-19 inequalities research: Who had the capacity to respond?

**DOI:** 10.1371/journal.pone.0266132

**Published:** 2022-05-12

**Authors:** Joan Benach, Lucinda Cash-Gibson, Diego F. Rojas-Gualdrón, Álvaro Padilla-Pozo, Juan Fernández-Gracia, Víctor M. Eguíluz, the COVID-SHINE group

**Affiliations:** 1 Research Group on Health Inequalities, Environment and Employment Conditions (GREDS-EMCONET), Pompeu Fabra University, Barcelona, Spain; 2 Johns Hopkins University-Pompeu Fabra University Public Policy Center (JHU-UPF PPC UPF-BSM), Barcelona, Spain; 3 Ecological Humanities Research Group (GHECO), Universidad Autónoma de Madrid, Spain; 4 Pompeu Fabra University–UPF Barcelona School of Management (UPF-BSM), Barcelona, Spain; 5 Faculty of Medicine, CES University, Medellín, Colombia; 6 Institute for Cross-Disciplinary Physics and Complex Systems, Campus Universitat de les Illes Balears, Palma de Mallorca, Islas Baleares, Spain; University of Central Florida, UNITED STATES

## Abstract

The COVID-19 pandemic has been testing countries’ capacities and scientific preparedness to actively respond and collaborate on a common global threat. It has also heightened awareness of the urgent need to empirically describe and analyze health inequalities to be able to act effectively. In turn, this raises several important questions that need answering: What is known about the rapidly emerging COVID-19 inequalities research field? Which countries and world regions have been able to rapidly produce research on this topic? What research patterns and trends have emerged, and how to these compared to the (pre-COVID-19) global health inequalities research field? Which countries have been scientifically collaborating on this important topic? Where are the scientific knowledge gaps, and indirectly where might research capacities need to be strengthened? In order to answer these queries, we analyzed the global scientific production (2020–2021) on COVID-19 associated inequalities by conducting bibliometric and network analyses using the Scopus database. Specifically, we analyzed the volume of scientific production per country (via author affiliations), its distribution by country income groups and world regions, as well as the inter-country collaborations within this production. Our results indicate that the COVID-19 inequalities research field has been highly collaborative; however, a number of significant inequitable research practices exist. When compared to the (pre-COVID-19) global health inequalities research field, similar inequalities were identified, however, several new dynamics and partnerships have also emerged that warrant further in-depth exploration. To ensure preparedness for future crises, and effective strategies to tackle growing social inequalities in health, investment in global health inequalities research capacities must be a priority for all.

## Introduction

Since the start of the COVID-19 pandemic, social and health inequalities have been exacerbated [1, 2]. Consequently, there has been a heightened awareness of the need to empirically describe and analyze these inequalities, and to report on the context-specific actions needed to effectively address them [3]. In many ways, the COVID-19 pandemic can be interpreted as a sort of natural experiment that has been testing every countries’ capacities and scientific preparedness to actively and effectively respond to such a socio-ecological-public health crisis, and to reduce its impact on citizens [4]. Countries need to know who is being affected, where, and in what magnitude. Yet, do all countries have the technical and human resource capacity to rapidly and effectively gather and comprehensively analyze health and socio-demographic data, and to develop comprehensive equity-oriented COVID-19 analyses [3]? Also, to what degree are countries collaborating to try to better understand the causes and effects of the pandemic on different social groups at the local, national and global level?

Prior to the pandemic, evidence demonstrated that global inequalities existed in this type of research capacity [5], which, as some scholars have already pointed out [3], has likely hindered many countries’ scientific capacity and preparedness to develop empirical evidence on COVID-19 inequalities, and effectively respond to the COVID-19 pandemic. Scientific production is considered to be a good proxy indicator of research capacity, as it is a comparable source of information that can indicate the volume of research that has been undertaken among different people and places, amongst other things [5]. However, what do we know about the emerging COVID-19 inequalities research field so far? Which countries and world regions have been able to rapidly produce research on this topic? What research patterns and trends have emerged in this new research field, and how do these compare to the global health inequalities research field? Which countries have been collaborating on this important scientific topic? Where are the scientific knowledge gaps, and, indirectly, where might related research capacities and research networks need to be strengthened? To try to answer these questions, and provide a first snapshot of this important and rapidly emerging research field, this study aims to analyze the volume of global scientific production on COVID-19 inequalities (2020–2021), its distribution by country income groups and world regions, as well as examine the inter-country collaborations. This snapshot can serve as a basis from which to conduct further in-depth analyses of COVID-19 inequalities research and related capacities at national level.

## Methods

### Study design

We conducted a bibliometric analysis and network analysis of original scientific articles on COVID-19 inequalities, published between January 2020 and April 23^rd^ 2021.

### Search strategy

Scientific articles were retrieved from the Scopus database, as this allows for bibliometric analysis and provides a larger access to multidisciplinary articles than other databases such as Web of Science [6], which is important given the fact that the health inequalities research field is known to be analysed from diverse scientific fields. In this study we use the term ‘health inequalities’ to refer to the following terms: health inequalities, health inequities, health disparities, and social inequalities in health. To ensure high sensitivity of our results, our search strategy combined a number of semi-free-text search terms to identify scientific articles on both COVID-19 and health inequalities, and built on the search strategy used in a previous (pre-COVID-19) bibliometric analysis on the global health inequalities research field (1966–2015), which also used Scopus, and considers the evolution of the terminology used about health inequalities across time and geography [5]. No geographical or language restrictions were applied. The following search strategy, specific to SCOPUS, was used:

[Title-abstract-keyword]: ((covid* PRE/3 *19) OR (*19 PRE/3 covid*) OR (coronavirus PRE/3 *19) OR (*19 PRE/3 coronavirus) OR ("novel coronavirus") OR ("new coronavirus") OR ("SARS-CoV-2") OR ("2019-nCoV")) AND ((inequ*) OR (dispari*) OR (equit*) OR (equal*) OR (povert*) **OR** (*igual*) OR (“pobreza”) OR (equid*) OR (*soci* PRE/2 gradient) **OR** (“gender”) **OR** (“género”) OR (ethni*) OR (“race”) OR (“racism”) **OR** (social PRE/2 class) OR (prejud*) **OR** (marginal*) OR (refugee*) OR (migrant*) OR (“homeless”) OR (“housing”) OR (“precarious”) OR (unemploy*))

It is worth noting that in SCOPUS, the semi-fixed term “PRE/n” means that the first word must be no more that (n) words apart from the next word.

### Data processing

We exported the metadata, including the citation and bibliographical information of the documents, their abstracts, keywords, and references. We calculated bibliometric variables by country, according to authors’ country of affiliation: the number of co-authored articles, the number of correspondence author articles, and the number of scientific collaborations for each possible pair of countries (i.e. country is the unit of analysis).

To standardize research output by country size, we calculated rates per country population, the Country Research Production (CRP) rate is defined as the number of co-authored articles per million habitants. The country correspondence author rate is defined as the number of correspondence authors per million habitants. We obtained the most recent estimation of the population from the World Bank Database [7]. We also followed the World Bank´s classification to classify countries into country income groups and world regions.

### Data analysis

Bibliometric analyses were performed with the Bibliometrix 3.1.3 [8] package in R 4.1.0. Statistical analyses were performed in Stata 17.0. The analysis of Gini coefficients was performed using our own code in Python.

We describe the research output, the average citations per year per article, the average number of authors per article, and the collaboration index: the average number of authors per article considering only multi-authored articles [8]. The landscape of international research collaborations within the COVID-19 inequalities research field was studied by a joint analysis of the research output, and collaborations based on Louvain’s clustering algorithm only considering countries with a minimum of 10 collaborations with other countries.

**Fig 1:**
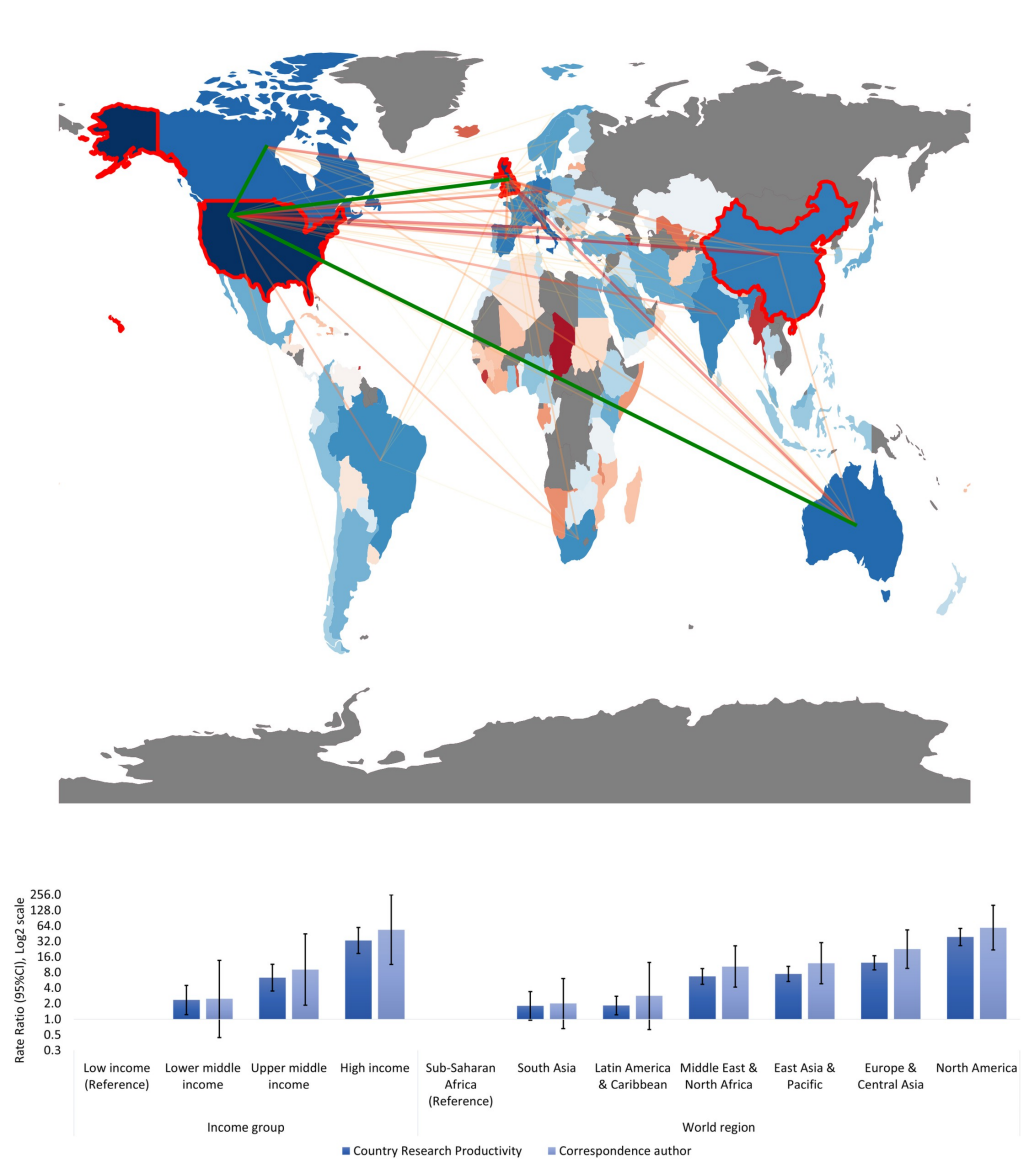
Geographical distribution (A) and inequalities in COVID-19 inequalities scientific production and co-author collaborations (B). (A) Choropleth map of the world showing the different degrees of collaborations between countries in this research field. The color of the country reflects the number of articles in collaboration with other countries (increasing from red to blue). The color of the connections encodes the number of articles in which the two linked countries appear (increasing from yellow to red; with the green connection indicating the ´highest producing´ research cluster). (B) Country Research Productivity (CRP) and Correspondence author rates per million people by country income group and world region.

A choropleth map was created to demonstrate the geographical distribution of the co-author collaborations within the COVID-19 inequalities scientific output (2020–2021) (Fig 1A). The color of the country reflects the number of articles in collaboration with other countries (increasing from red to blue), and the color of the connections encodes the number of articles in which the two linked countries appear (increasing from yellow to red). Only connections with more than 20 collaborations are shown. The map was produced using the package geopandas in Python with our own code.

The differences in COVID-19 inequalities research between countries, according to income group and world region, were quantified with productivity rate ratios (RR). The generalized linear model with Poisson distribution and logarithmic link function was used to estimate RR and 95% confidence intervals (95% CI) (Fig 1B).

**Fig 2:**
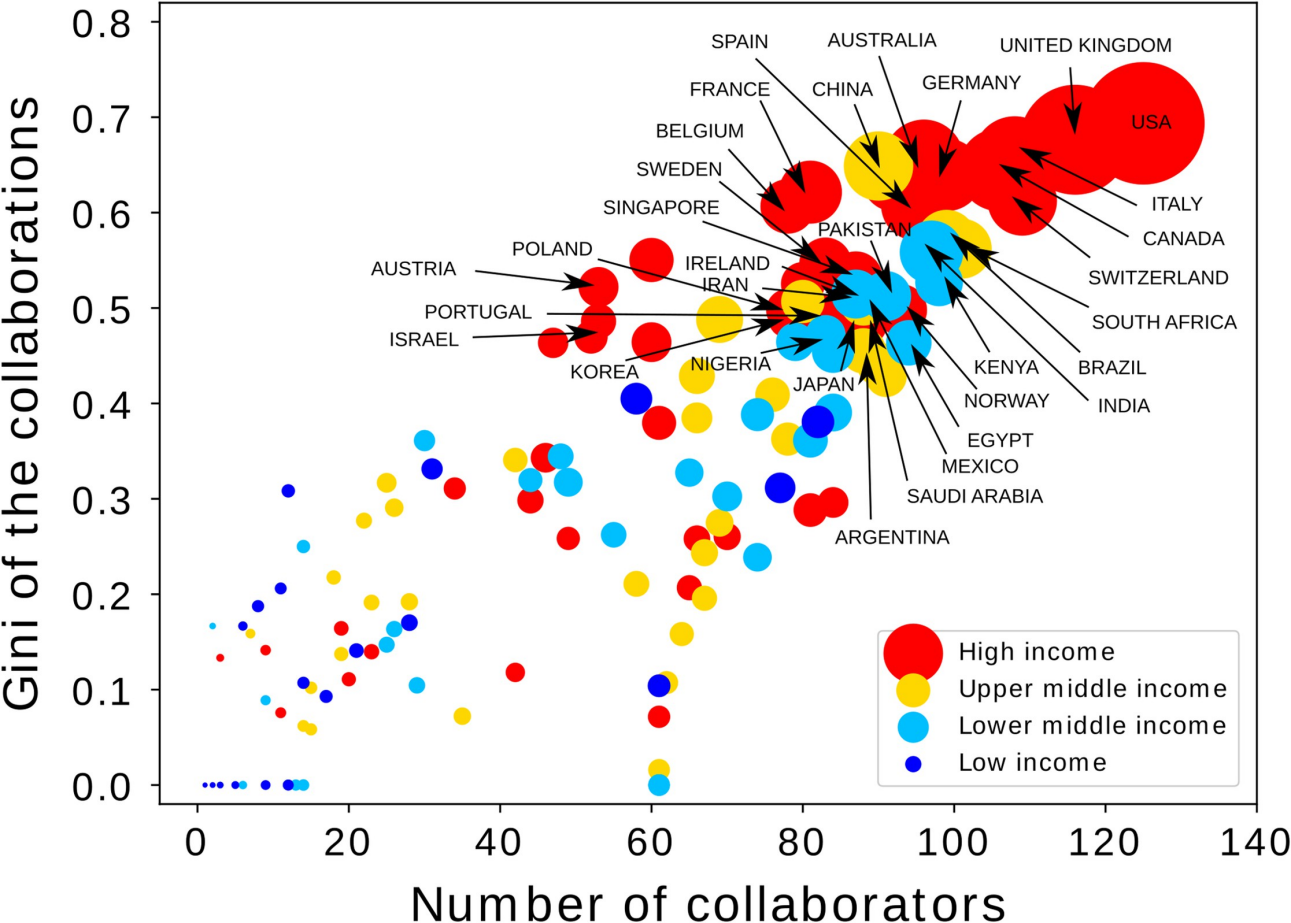
Gini coefficient between international collaborators, as a function of the number of collaborators per country. Size of the symbols reflects the number of articles for each country in collaboration with another country. Color indicates the country income group. Only 30 countries with the highest Gini, and the highest number of collaborations are shown.

For each country we also calculated the Gini coefficient (the degree on inequality) for the distribution of articles in collaboration with other countries. The analysis was performed using Python. The value of the Gini coefficient is shown as a function of the number of collaborators (Fig 2).

**Fig 3:**
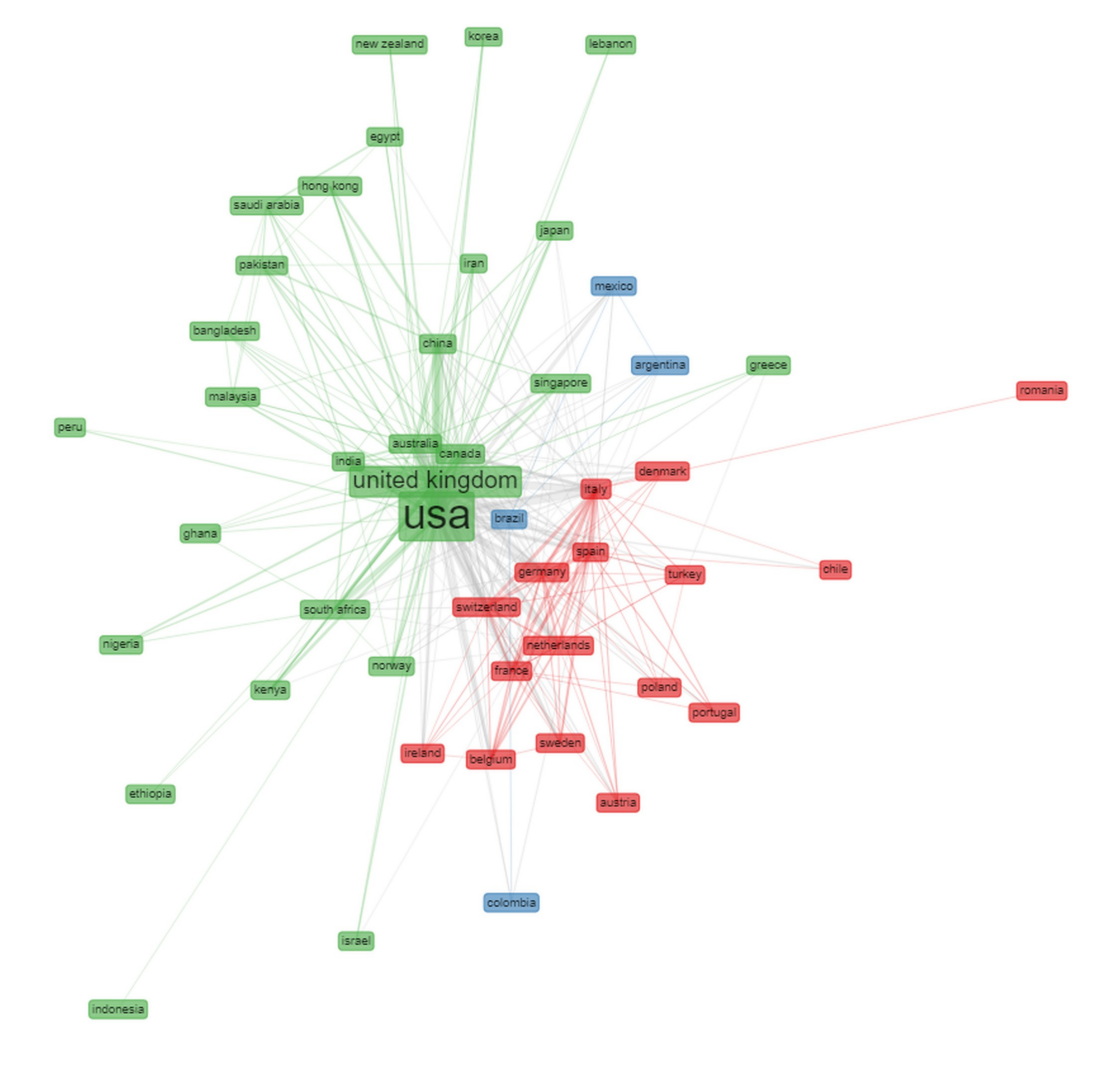
Clusters of co-authorship networks of COVID-19 inequalities research (2020–2021). The network visualisation map depicts the publications co-authored by each country of affiliation relating to the individual countries link strength network (i.e. inter-country co-author relation) within these clusters. Each color represents a distinct research cluster, and the size of each rectangle represents the countries’ total scientific production on COVID-19 inequalities.

Finally, we created a bibliometric network visualization map using bibliometrix, which depicted the publications co-authored by each country of affiliation relating to the individual countries link strength network (i.e. inter-country co-author relation) within these clusters. Only countries with a minimum of 10 collaborations were included (Fig 3). (See S1 File for supporting information for the figures).

## Results

Our search identified 9,355 original scientific articles, with 140 countries (via authors country affiliation) having contributed to at least one article. The three highest country producers of COVID-19 inequalities research were the United States (US) (32% of the total scientific production), the United Kingdom (UK) (10%), and China (6%) (Fig 1A). In addition, our results found that the country contributions to this new research field were highly unequal between and within different country income groups and world regions (Figs 1A, 1B, and 2). High-income countries (HIC) showed a CRP rate 33 times higher and a correspondence author rate 54 times higher than low-income countries (LIC) (Fig 1B). These differences were smaller, though still substantial, for upper and lower middle-income countries (MIC), whose CRP rates were six and three times higher, and correspondence author rates were nine and two times higher, respectively, than that of LIC. As for the inequalities between world regions, in comparison to Sub-Saharan Africa, North America’s CRP rate and correspondence author rate were 39 and 59 times higher, Europe’s were 12 and 23 times higher, the Middle East & North Africa’s were seven and 12 times higher, East Asia & the Pacific’s were seven and ten times higher, South Asia’s were two and three times higher, and Latin America & Caribbean’s were two times higher (Fig 1B).

The scientific production on COVID-19 inequalities (2020–2021) has been highly collaborative, 84.5% of the articles (n = 7,902) had multiple co-authors, and had a collaboration index of 5.5. Nevertheless, inequalities were also present within these collaborations: we found a positive relationship between the number of collaborators of a country and the Gini coefficient (i.e. the degree of inequality) between collaborators (Fig 2). In addition, authors affiliated to HIC institutions have contributed to more of this scientific production and to the international research collaborations than author’s affiliated to MIC or LIC institutions (Fig 1B).

Three main international collaboration network clusters were identified within the scientific production (Figs 1A and 3). One (green) cluster was formed mainly by Anglosaxon (e.g. the UK, US, Canada, Australia), Asian and African countries; a second (red) cluster was formed mainly by European countries, and Chile, and a third (blue) cluster was formed by four Latin American countries, namely Colombia, Brazil, Mexico and Argentina. The US and UK were the most predominant countries within the global research collaborations.

## Discussion

The COVID-19 inequalities research field has rapidly emerged and expanded over the past year (2020–2021), and has been highly collaborative, indicating some degree of scientific cooperation on this topic, yet notable inequalities exist in the research productivity rates between countries, country income groups and world regions (Fig 1B), as well as within the research collaborations (Fig 2). Our results identified the US, UK and China as the three highest country producers of COVID-19 inequalities research (2020–2021) (Fig 1A). These findings are in line with the results of a recent bibliometric analysis of the global scientific research on COVID-19 [9]. Together these three countries have produced 48% of the total COVID-19 inequalities scientific production (2020–2021).

Many of our findings also concur with those of a recent, albeit pre-COVID-19, bibliometric analysis of the global health inequalities research field (1966–2015) [5], which identified a number of inequalities within the scientific production and international research collaborations. Firstly, our results found that in the COVID-19 inequalities scientific production (2020–2021) HIC had a research productivity rate 33 times higher than that of LIC (Fig 1B). Similar results were observed in the bibliometric analysis of the global health inequalities research field [5] which analysed the time trends in the global scientific production on health inequalities over five decades by country income group, and found a visible country-income group affiliation gradient in the initiation and consistent publication frequency on this topic. This long standing country-income group affiliation gradient therefore appears as a reproduced pattern in this emergent related research field, and influenced the capacity to rapidly conduct and produce research on COVID-19 associated inequalities in many countries around the world. Secondly, the largest regional producer of COVID-19 inequalities research is North America, followed by Europe and Central Asia, then the East Asian and the Pacific region (Fig 1B). Thirdly, the US and UK are still the most predominant collaborators, and the two highest country producers of this research, and there is still also a notably predominant European collaborative research cluster (Figs 1A and 3).

Interestingly though, when comparing these two research fields we also identified a number of differences in the research patterns and trends, which are worth noting. Firstly, the US is at the center of all the collaborative networks in the COVID-19 inequalities research field, followed by the UK, as opposed to the UK being at the center of the collaborations [5]. Secondly, a new (blue) Latin American cluster has emerged consisting of four countries; these countries were previously identified as the four highest producers of health inequalities research in the Latin American and Caribbean region [5, 10], and are known to have had a tradition of collective health and social medicine research [10, 11], which indicates a strong health inequalities research capacity within the region [5]. This has likely been important to enable them to rapidly conduct and produce research on COVID-19 inequalities. Curiously, Chile does not form part of this cluster, although our findings indicate that it has retained strong inequality-related research collaborations with Brazil. Rather, Chile forms part of the predominantly “European” (red) cluster, this may partly be due to the fact that there were pre-existing collaborations between research groups working on health inequalities within Spain and Chile, for example, particularly around the so-called “…s*ocio-critical accounts of work-related and migrant health”* [12] (p.5), which have likely been leveraged during the pandemic. Thirdly, our study found that the Middle Eastern and North African region has a high productivity rate and correspondence author rate, and is the fourth largest regional producer of COVID-19 inequalities research, almost on par with the East Asian and the Pacific region, and higher than that of the Latin America and Caribbean region. When compared with the global health inequalities research field [5], the Middle Eastern and North African region´s had previously been one of the lowest regional scientific producers on health inequalities.

The Middle Eastern and North African region had its own distinct health inequalities research cluster [5]; however, our findings identified several countries from the region which now form part of the predominantly “Anglosaxon” (green) COVID-19 inequalities research cluster. This may be due in part to the fact that in 2019 a new Commission on Social Determinants of Health was established in the WHO Eastern Mediterranean region, led by Sir Michael Marmot and his team at the Institute of Health Equity, University College London, UK, and in collaboration with the World Health Organization´s Alliance for Health Policy and Systems Research, Switzerland. The Commission´s objectives have been to analyze health inequalities across the region, generate relevant knowledge and evidence, and develop context-specific recommendations to reduce health inequalities [13]. Given that the pandemic occurred shortly afterwards the Commission was established, research on COVID-19 related inequalities has been conducted by the team [14], which likely explains these newly formed international research collaborations identified.

Bibliometric analysis is an extremely useful quantitative tool that is being increasingly utilised in different disciplines to measure and evaluate trends in scientific output, and its findings can support evidence informed decision-making [5, 15]. There has been a bibliometric analysis on the global COVID-19 research field more broadly [9]; however, given the importance of the topic of COVID-19 associated inequalities in these new times, and the fact that this field of research is distinct from the broad COVID-19 research and from the (pre-COVID-19) global health inequalities research, it therefore warrants its own analysis.

While our results are based only on empirical articles published in international academic journals, they provide a useful overview of the likely global dynamic and patterns within this potentially new scientific field. In addition, as scholars have noted [5], determining a countries proportional contribution to this research field by income group and world regions, enables a better understanding the global research landscape, and allows for potentially ´fairer´ country and regional comparisons to be made, as well as for many more subsequent research questions to be posed.

In our study, the unit of analysis was ´country´, as author-affiliation provides a proxy indication of a country’s contribution to the research field, and their capacity to conduct this type of research, which is the main interest of our study. As previous bibliometric analyses note [5], author-affiliation does not necessarily represent the original nationality of the author; however, it is the best proxy indication available for country contributions. For example, if an author signs their affiliated to a certain institution within a certain country, then the assumption is that they are a member of that countries scientific community. We did not assess the country of study within the COVID-19 inequalities articles, given that a researcher from any country can study a topic within any country, and does not necessarily need to be associated to that countries scientific community, or to include researchers from the country under investigation. This often occurs within global (or previously international) health research, where neo-colonial research practices and ´epistemic injustice´ [16–19] are well known to exist. Therefore, this particular assessment would not provide any indication of the research capacity of the country under investigation, only of the country investigating it. Furthermore, given the long-standing inequitable research practices, this is why it was important to analyse, and demonstrate the degree of inequality within the global scientific collaborations on COVID-19 inequalities.

Further research is needed to establish more in depth understanding of why and how these trends, dynamics and outcomes might have occurred in the COVID-19 inequalities research field, and of the context factors and determinants of these research capacities at institutional, local, and national level, as well as the key mechanisms that may be activated or inhibited to generate this scientific output [20–23]. Also, assessments of the capacity to produce COVID-19 inequalities research at national level could help identify any strengthens, challenges or information gaps, and assist to guide future strategies that aim to further strengthen these capacities [3, 20, 21], to ensure better scientific preparedness for future crises. This should include an assessment of the relationship between productivity research rates by country, and other variables of interest, such as the share of the GDP invested in health and social sciences research and development [5], as well as the type of COVID-19 inequalities research produced in different countries–both the content of the research, as this may also indicate priority areas for action at national level, as well as the different disciplinary and political perspectives that frame the research [3, 21]. Lastly, as the pandemic continues and evidence on COVID-19 inequalities grows, it will be useful to conduct regular, similar analyses to monitor these research trends and dynamics over time.

The COVID-19 pandemic has been testing countries’, communities and individuals in many ways. At country level, political and scientific capacities, solidarity and preparedness to actively and effectively respond to such a global crisis have been under scrutiny. It is important to note that these capacities, and the type of policy responses taken, are conditioned by a number of pre-existing contextual factors, such as the level of pre-existing health inequalities, politics, ideology and value perspectives on health inequalities, and what each society considers to be socio-politically desirable (e.g., collectivism versus individualism) [3, 20, 24]. In addition, country’s economic resilience, public trust in government, and general trust in science has also played a role [3, 25–27].

Collectively, what is clear is the need for every country to develop stronger capacity to collect timely, reliable, disaggregated by different social groups (e.g., social class, age, gender, ethnicity/race, geography), to establish comprehensive COVID-19 data collection systems to be able to regularly report on, and monitor health inequalities [3, 13, 20, 28–30]. In addition, while research networks can help to mobilize and share resources, they also create new conditions, dynamics and power relations which are brought into the research production process which need to be considered [20, 21]. Moving forward, ethical principles must be instilled in research collaborations to ensure more equitable global health research partnerships, which is another important, growing research field in itself [20, 31–34].

Being able to rapidly conduct comprehensive COVID-19 inequalities analyses can assist to produce vital knowledge about COVID-19 related incidences, deaths, and health inequalities in the community [3], as well among health professionals [35–38]. This knowledge is needed to help guide the development of more reactive, evidence-informed policy and action aiming to address COVID-19 related inequalities, and raise general awareness of these issues at local, national, and global levels to ensure a healthier future for all.

## Conclusion

Bibliometric and network analyses are extremely useful tools, which have enabled us to provide a first snapshot of the rapidly emerging COVID-19 inequalities research field, including its trends and dynamics, and research practices. Our study found this new research field to be highly collaborative; however, inequitable research practices exist, and when compared to the (pre-COVID-19) global health inequalities research field, new dynamics have emerged that warrant further in-depth exploration. This snapshot can serve as a basis from which to pose further research questions, and to conduct assessments of local COVID-19 inequalities research capacities, to identify strengthens and areas for improvement. To ensure better preparedness for future crises, and more effective strategies to tackle health inequalities and achieve a healthy future for all, investment in health inequalities research capacities must be a local and global priority.

## Supporting information

S1 File Supporting information for figures. (ZIP)
