# Supplementary material for: Inequalities in COVID-19 inequalities research: Who had the capacity to respond?
Source: PLoS One. 2022 May 12;17(5):e0266132. doi: 10.1371/journal.pone.0266132 (PMC9098009; doi:10.1371/journal.pone.0266132)
Supplement: S1 File — (ZIP) [file pone.0266132.s001.zip › data_and_code_supplementary file.zip!/data_and_code/###readme.rtf]

Information about the files included in the folder

“Wold_collaboration_map.csv” contains the data needed to plot Figure 1A
“plot_map_bibliometric.py”	contains the code to that computes Figure 1A in Python

“1B_data.xlsx” contains the data needed to plot Figure 1B
“1B_code.R” contains the code used to obtain the estimates used to plot Figure 1B in R

“income_groups.csv” contains the data used to calculate the Gini coefficient in Figure 2
“analyze_network.py” contains the code to compute Figure 2 in Python
